# Supplementary material for: Common origin of sterol biosynthesis points to a feeding strategy shift in Neoproterozoic animals
Source: Nat Commun. 2023 Dec 1;14:7941. doi: 10.1038/s41467-023-43545-z (PMC10692144; doi:10.1038/s41467-023-43545-z)
Supplement: Supplementary file 1 — Supplementary Information [file 41467_2023_43545_MOESM1_ESM.pdf]

SUPPLEMENTARY INFORMATION

SUPPLEMENTARY METHODS

Fossil Calibrations. Nine fossil calibration points were selected to calibrate our molecular clock analysis, following best practices [54]. Where possible, calibration points were confirmed using the Paleobiology Database (PBDB; https://paleobiodb.org). Calibration priors were modeled as lognormal distributions with a sigma of 2.5, and an offset using the minimum age of the fossil. Such priors place the highest confidence for the true date of nodes to be slightly older than the fossil, with decreasing probabilities extending deep into the past.

Clade Annelida (stem)
Minimum: 558.3 Ma
Median: 559.3
95% Confidence Interval: 619.4 Ma
97.5% Confidence Interval: 692.6 Ma
Taxa included: *Alitta virens; Amynthas gracilis; Capitella teleta; Eisenia fetida; Enchytraeus albidus; Enchytraeus crypticus; Glossoscolex paulistus; Lamellibrachia luymesi; Lamellibrachia satsuma; Lumbriculus variegatus; Lumbricus castaneus; Lumbricus rubellus; Olavius algarvensis; Ophryotrocha diadema; Paraescarpia echinospica; Perinereis aibuhitensis; Platynereis dumerilii; Pygospio elegans; Romanchella perrieri; Spirobranchus lamarcki*

The fossil *Sabellidites cambriensis*, an Ediacaran annelid from the Nekrasovo Formation, Russia, was used in this dataset to calibrate all Annelida [55]. The age of this taxa is constrained by dated ash beds both below and comprising the formation (the later dated to 555.3 and 558.3 Ma, respectively). The specimen (ME-Pl-1-30) is kept in the Museum of Evolution, at Uppsala University, Sweden. Morphologically, chemically, and microstructurally the tubes of this fossil support a siboglonid affinity, but a recent paper [56] argued that many of these characteristics do not correlate to modern clades. Therefore, we used this taxon to calibrate the stem group Annelida.

Clade Bikonta
Minimum: 1047 Ma
Median: 1048 Ma
95% Confidence Interval: 1108 Ma
97.5% Confidence Interval: 1181 Ma
Taxa included: *Arabidopsis thaliana; Aureococcus anophagefferens; Chlamydomonas reinhardtii; Emiliania huxleyi; Grammatophora oceanica; Guillardia theta; Heterocapsa*

rotundata; Karenia mikimotoi; Odontella aurita; Physcomitrium patens; Symbiodinium cladeA; Symbiodinium cladeD; Thalassiosira pseudonana; Volvox carteri

The fossil *Bangiomorpha pubescens*, collected from the Hunting Formation of Somerset Island, Canada, has been dated to 1.047 Gy [57]. It is widely considered a red algae; defining traits include filaments with fourfold radial symmetry and regular intercalary radial division [58]. Because red algae are more closely related to *Bangiomorpha* than other algae in our dataset, we can use *Bangiomorpha* for a crown age calibration.

Clade Coccolithophore
Minimum: 201.3 Ma
Median: 202.3 Ma
95% Confidence Interval: 262.4 Ma
97.5% Confidence Interval: 335.6 Ma
Taxa included: *Emiliania huxleyi; Guillardia theta*

The fossil *Crucirhabdus minutus* was chosen to calibrate the clade Coccolithophoreas. It was first described from different localities in Austria, northern Italy, and southern Germany [59]. We used the oldest stratigraphic occurrence of *C. minutus* from the Pötschen Fm. of the Northern Calcareous Alps of Austria to calibrate Coccolithophore [60]. This fossil is therefore used as a crown calibration to separate the cryptophyte *Guillardia theta* from the coccolithophore *Emiliania huxleyi*.

Clade Embryophyta (stem)
Minimum: 419.2 Ma
Median: 420.2 Ma
95% Confidence Interval: 480.3 Ma
97.5% Confidence Interval: 553.5 Ma
Taxa included: *Arabidopsis thaliana; Physcomitrium patens*

The plant fossil *Cooksonia pertoni* (PBDB collection #: 10593 & 10706), collected from the Rushall Formation (Fm.) (alternatively the "Downton Castle Sandstone Fm.) of the Welsh Borderland, England was used to calibrate the Embryophyta. *Cooksonia pertoni*, was originally named and described as a plant but later assigned to the Psilophytales [61,62]. *Cooksonia pertoni* was classified as a vascular plant based on well-preserved smooth and autonomously branching axes, solitary terminal sporangia, stomata, sterome, and also thick-walled spores [63]. We used *C. pertoni* to calibrate stem group Embryophyta, with an age minimum of 419.2 Ma estimated from ostracods and spores [64].

Clade Fungi (crown)
Minimum: 407.6 Ma
Median: 407.6 Ma
95% Confidence Interval: 468.7 Ma
97.5% Confidence Interval: 541.9 Ma
Taxa included: *Puccinia graminis; Saccharomyces cerevisiae*

Fossil ascomycete from the 400 million-year-old Rhynie chert was used to calibrate the Fungi clade. The Rhynie chert fossil fungi assemblage represents the oldest robust fossil fungi owing to well preserved vegetative mycelium and chlamydospores [65]. Fossil fungi from the Dryden Fm. were initially reported based on the presence of well preserved reproductive organs of saprophytic fungi but were not formally described in detail or named until 1921 by the same authors [65,66]. These specimens are housed in the Hunterian Museum and in the Geological Collections of the Bristol Museum and Art Gallery, collection BRSGM. The fossil fungi of the Rhynie chert have been corroborated by numerous subsequent studies [67] (see references from Krings et al., 2018). First reports of the oldest ascomycete are based on well-preserved perithecia and asci, the sac-like structure where the sexual ascospores are produced. Ascomycota contains the taxon *Saccharomyces cerevisiae*, therefore their oldest fossil representative is used to calibrate crown group fungi. The age minimum of 407.6 was established based on $^{40}Ar/^{39}Ar$ in K-feldspar [68].

Clade Hesionoidea (stem)
Minimum: 300 Ma
Median: 301 Ma
95% Confidence Interval: 361.1 Ma
97.5% Confidence Interval: 434.3 Ma
Taxa included: *Alitta virens*; *Perinereis aibuhitensis*; *Platynereis dumerilii*

The superfamily Hesionoidea comprises the clades Chrysopetalidae and Hesionidae as recovered in a recent genomic analysis [49]. *Rutellifrons wolfforum*, a Carboniferous annelid from the Mazon Creek area of Illinois [69], was chosen to calibrate this clade. *R. wolfforum* is considered hesonoid based on internal asiculae and compound chaetae [70]. Cladistic analysis supports the placement of *R. wolfforum* in the Phyllodocida, consistent with a stem-Hesionid assignment [71]. The exact age range for the Mazon Creek area has been contested over the years [72], so the upper limit of the Pennsylvanian (300 Ma) was chosen.

Clade Petrosiidae (stem)
Minimum: 38 My
Median: 39 My
95% Confidence Interval: 99.08 My
97.5% Confidence Interval: 172.3 My
Taxa included: *Amphimedon queenslandica; Haliclona tubifera; Neopetrosia compacta*

The demosponge family Petrosiidae van Soest (1980) (Demospongiae, Haplosclerida) was calibrated using the fossil *Propetrosia pristina* (PBDB collection number: 61247) from the La Guixa Member (Mb.) of the Vic Marls Formation (Fm.) in Catalonia, Spain. The materials are housed at the Institute of Paleobiology, Polish Academy of Sciences, Warszawa, under the collection number ZPAL Pf.X, and in the Museum Geologic del Seminari, Barcelona, collection acronym MGSB. *Propetrosia pristina* was initially described and assigned to the family Petrosiidae, order Haplosclerida, based on typical haplosclerid spiculation and structure [73]. Specifically, an outer surface consisting of a relatively thick layer of multispicular, closely arranged crust of bundled and unbundled oxeas spicules forming circular structures [73]. *Propetrosia* was later confirmed as belonging to the order Haplosclerida [74]. *Propetrosia pristina* is an extinct species of demosponge and therefore used as a stem-group calibration. We used the minimum age of 38.0 Ma for the fossil-based on data retrieved from the PBDB.

Clade Sabellida (stem)
Minimum: 265.1 Ma
Median: 266.1 Ma
95% Confidence Interval: 326.2 Ma
97.5% Confidence Interval: 399.4 Ma
Taxa included: *Lamellibrachia luymesi; Lamellibrachia satsuma; Paraescarpia echinospica; Romanchella perrieri; Spirobranchus lamarcki*

The clade Sabellida is calibrated with the fossil taxa *Glomerula testatrix* and *G. gemmellaroi*, both from the Sosio Valley, Italy [75]. The oldest age of the limestone deposits is Wordian (268.8-265.1 Ma), which is largely based on ammonite and foram stratigraphy [76]. The fossil *Glomerula* species are associated with this clade based on glomerate coiling, slow tube expansion, and the absence of basal cementing flanges. Given that this genus has extant members, the fossil taxa are considered crown group Sabellida for this analysis. *G. testatrix* specimens (MSNC4515 through MSNC4518) and G. *gemmellaroi* (MSNC4519 through MSNC4523) are housed at the Museo Civico di Storia Naturale di Comiso, Ragusa, Italy.

Clade Scleractinia (stem)
Minimum: 452 Ma
Median: 453 Ma
95% Confidence Interval: 513.1 Ma
97.5% Confidence Interval: 586.3 Ma
Taxa included: *Agaricia lamarcki; Astroides calycularis; Cyphastrea serailia; Favites colemani; Galaxea fascicularis; Orbicella faveolata; Porites astreoides; Porites australiensis; Porites lutea; Siderastrea siderea*

The cnidarian order Scleractinia was calibrated using the fossil *Kilbuchophyllum discoidea* (Anthozoa, Kilbuchophyllida) (PBDB collection #: 123174) from the Kirkcolm Fm. in the Southern Uplands of Scotland. Fossil materials are housed in the Royal Museum of Scotland, Edinburgh (RMS). The fossil was originally described and assigned to the new family Kilbuchophyllidae, and the new order Kilbuchophyllida [77]. We used *Kilbuchophylllum discoidea* to calibrate stem group Scleractinia based on the clear expression of cyclic, hexameral septa, a defining characteristic of Scleractinia [77]. The age minimum of 452 Ma is based on data retrieved from the PBDB, given the fossil's proximity to the Soudleyan-Actonian boundary (457-458 Ma) [77].

SUPPLEMENTARY REFERENCES

54. Parham, J. F. *et al.* Best practices for justifying fossil calibrations. *Systematic Biology* **61**, 346–359 (2012).
55. Moczydłowska, M., Westall, F. & Foucher, F. Microstructure and Biogeochemistry of the Organically Preserved Ediacaran Metazoan Sabellidites. *Journal of Paleontology* **88**, 224–239 (2014).
56. Georgieva, M. N. *et al.* Identification of fossil worm tubes from Phanerozoic hydrothermal vents and cold seeps. *Journal of Systematic Palaeontology* **17**, 287–329 (2019).
57. Gibson, T. M. *et al.* Precise age of Bangiomorpha pubescens dates the origin of eukaryotic photosynthesis. *Geology* (2017).
58. Butterfield, N. J. Bangiomorpha pubescens n. gen., n. sp.: implications for the

evolution of sex, multicellularity, and the Mesoproterozoic/Neoproterozoic radiation of eukaryotes. *Paleobiology* **26**, 386–404 (2000).

59. Jafer, S. A. Significance of Late Triassic calcareous nannoplankton from Austria and southern Germany. *Neues Jahrbuch fur Geologie und Palaontologie. Abhandlungen* **166**, 218–259 (1983).
60. Gardin, S., Krystyn, L., Richoz, S., Bartolini, A. & Galbrun, B. Where and when the earliest coccolithophores? *Lethaia* **45**, 507–523 (2012).
61. Andrews, H. N. *Index of generic names of fossil plants, 1820-1965*. vol. 1300 (United States Department of the Interior, Geological Survey, 1970).
62. Lang, W. H. On the plant-remains from the Downtonian of England and Wales (1937) Philosophical Transactions of the Royal Society of London. *B227* 245–291.
63. Edwards, D., Davies, K. L. & Axe, L. A vascular conducting strand in the early land plant Cooksonia. *Nature* **357**, 683–685 (1992).
64. Fanning, U., Richardson, J. B. & Edwards, D. Cryptic evolution in an early land plant. *Evolutionary Trends in Plants (ETP)* **2**, 13–24 (1988).
65. Kidston, R. & Lang, W. H. XXXIII.—On Old Red Sandstone plants showing structure, from the Rhynie Chert Bed, Aberdeenshire. Part V. The Thallophyta occurring in the peat-bed; the succession of the plants throughout a vertical section of the bed, and the conditions of accumulation and preservation of the deposit. *Earth and Environmental Science Transactions of the Royal Society of Edinburgh* **52**, 855–902 (1921).
66. Kidston, R. & Lang, W. H. XXIV.—On Old Red Sandstone Plants showing Structure, from the Rhynie Chert Bed, Aberdeenshire. Part II. Additional Notes on Rhynia

Gwynne-Vaughani, Kidston and Lang; with Descriptions of Rhynia major, n. sp., and Hornea Lignieri, ng, n. sp. *Earth and Environmental Science Transactions of the Royal Society of Edinburgh* **52**, 603–627 (1920).

67. Krings, M., Harper, C. J. & Taylor, E. L. Fungi and fungal interactions in the Rhynie chert: a review of the evidence, with the description of Perexiflasca tayloriana gen. et sp. nov. *Philosophical Transactions of the Royal Society B: Biological Sciences* **373**, 20160500 (2018).
68. Mark, D. F. *et al.* 40Ar/39Ar dating of hydrothermal activity, biota and gold mineralization in the Rhynie hot-spring system, Aberdeenshire, Scotland. *Geochimica et Cosmochimica Acta* **75**, 555–569 (2011).
69. Thompson, I. Errant polychaetes (Annelida) from the Pennsylvanian Essex fauna of northern Illinois. (1979).
70. Merz, R. A. & Woodin, S. A. Polychaete chaetae: function, fossils, and phylogeny. *Integrative and comparative biology* **46**, 481–496 (2006).
71. Parry, L., Tanner, A. & Vinther, J. The origin of annelids. *Palaeontology* **57**, 1091–1103 (2014).
72. Clements, T., Purnell, M. & Gabbott, S. The Mazon Creek Lagerstätte: a diverse late Paleozoic ecosystem entombed within siderite concretions. *Journal of the Geological Society* **176**, 1–11 (2019).
73. Pisera, A. & Busquets, P. Eocene siliceous sponges from the Ebro Basin (Catalonia, Spain). *Geobios* **35**, 321–346 (2002).
74. Rigby, J., Finks, R. M., Reid, R. E. H. & Kaesler, R. L. *Porifera: Demospongea, Hexactinellida, Heteractinida, Calcarea*. (2004).

75. Sanfilippo, R., Rosso, A., Reitano, A. & Insacco, G. First record of sabellid and serpulid polychaetes from the Permian of Sicily. *Acta Palaeontologica Polonica* **62**, 25–38 (2017).

76. Flügel, E., Di Stefano, P. & Senowbari-Daryan, B. Microfacies and depositional structure of allochthonous carbonate base-of-slope deposits:. The Late Permian Pietra di Salomone Megablock, Sosio Valley (Western Sicily). *Facies* 147–186 (1991).

77. Scrutton, C. T. A new scleractinian-like coral from the Ordovician of the Southern Uplands, Scotland. *Palaeontology* **34**, 179–194 (1991).

SUPPLEMENTARY FIGURES

Supplementary Figure 1. A phylogeny of SMTs produced by all putative SMT and SMT-like sequences. Sequences that were removed in downstream analyses are highlighted and annotated. The original tree file is provided as "9_SMT_Vetted.fas.contree.tree" on the manuscript's GitHub repository.

Supplementary Figure 2. **A phylogeny of vetted SMT sequences, before gene tree / species tree reconciliation with NOTUNG.** The original tree file is provided as "9_SMT_Vetted.fas.contree.tree" on the manuscript's GitHub repository.

Supplementary Figure 3. **A phylogeny of vetted SMT sequences after gene tree / species tree reconciliation with NOTUNG.** The original tree file is provided as "10_SMT_Gene_Tree.tree.rearrange.0.tree" on the manuscript's GitHub repository.

Supplementary Figure 4. Mapping of the *sterol methyltransferase* gene onto the *Porites australiensis* genome. The *smt* gene comes from the National Center for Biotechnology Information (NCBI) accession number FX438716.1; the genome comes from accession GCA_022179025.1. The original mapping file is provided as "2_Pau_mRNA.genome.gff" on the manuscript's GitHub repository.

Supplementary Figure 5. The full molecular clock that Figure 5 is based on. 95% confidence intervals are visualized with purple bars. The pink bar highlights the 95% confidence interval for the origin of eumetazoan *smt* genes. The red bar highlights the

95% confidence interval for the origin of bilaterian *smt* genes. The tree can be reproduced by running the file “11_SMT_BEAST.xml” from GitHub in BEAST v.1.10.4. The tree file is available as file “12_SMT_BEAST.con.tree”

Supplementary Figure 6. A rerun of the molecular clock with a different topology, removing the ancestral animal gene duplication. 95% confidence intervals are visualized with purple bars. The pink bar highlights the 95% confidence interval for the origin of animal *smt* genes. The red bar highlights the 95% confidence interval for the origin of eumetazoan *smt* genes. The tree can be reproduced by running the file “11_SMT_BEAST_AltTopology.xml” from GitHub in BEAST v.1.10.4. The tree file is available as file “12_SMT_BEAST_AltTopology.con.tree”

Supplementary Figure 7. Spectra of the ergosterol peaks from our gene transformation experiment. (A) The ergosterol mass spectrum from $ERG6^{-}$ yeast with transformation of the *C. teleta smt*, relating to figure 4B in the main text. (B) The ergosterol mass spectrum from a standard mix, relating to Figure 4C in the main text.

Figure S1

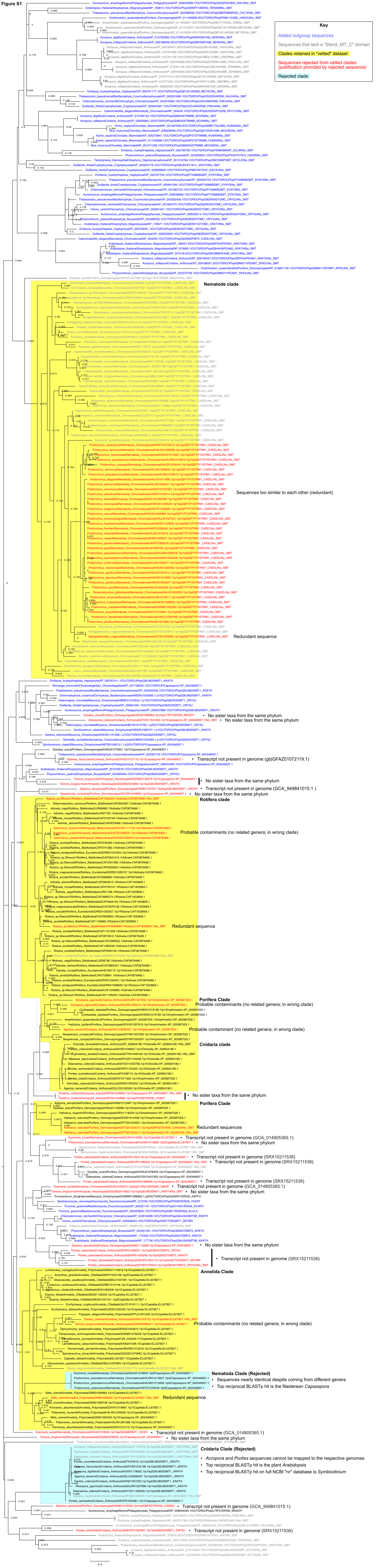

Key
Added outgroup sequences
Sequences that lack a "Sterol_MT_C" domain
Clades retained in "vetted" dataset
Sequences rejected from vetted clades (justification provided by rejected sequence)
Rejected clade
Nematode clade
Sequences too similar to each other (redundant)
Redundant sequence
No sister taxa from the same phylum
No sister taxa from the same phylum
Transcript not present in genome (gb|GFAZ01072119.1)
No sister taxa from the same phylum
Transcript not present in genome (GCA_949841015.1)
No sister taxa from the same phylum
Rotifera clade
Probable contaminants (no related genera; wrong clade)
Redundant sequence
Porifera Clade
Probable contaminants (no related genera; wrong clade)
Probable contaminant (no related genera; wrong clade)
Cnidaria clade
No sister taxa from the same phylum
Porifera Clade
Redundant sequences
Transcript not present in genome (GCA_014805365.1)
No sister taxa from the same phylum
Transcript not present in genome (SRX15211538)
Transcript not present in genome (SRX15211538)
Transcript not present in genome (SRX15211538)
Transcript not present in genome (GCA_014805365.1)
No sister taxa from the same phylum
Transcript not present in genome (SRX15211538)
Annelida Clade
Probable contaminants (no related genera; wrong clade)
Nematode Clade (Rejected)
Sequences nearly identical despite coming from different genera
Top reciprocal BLASTp hit is the filasterean Capsaspora
Redundant sequence
Transcript not present in genome (GCA_014865365.1)
No sister taxa from the same phylum
Cnidaria Clade (Rejected)
Acropora and Porites sequences cannot be mapped to the respective genomes
Top reciprocal BLASTp hit is the plant Arabidopsis
Top reciprocal BLASTp hit on full NCBI "nr" database is Symbiodinium
Transcript not present in genome (GCA_949841015.1)
Transcript not present in genome (SRX15211538)
0.4

Figure S2

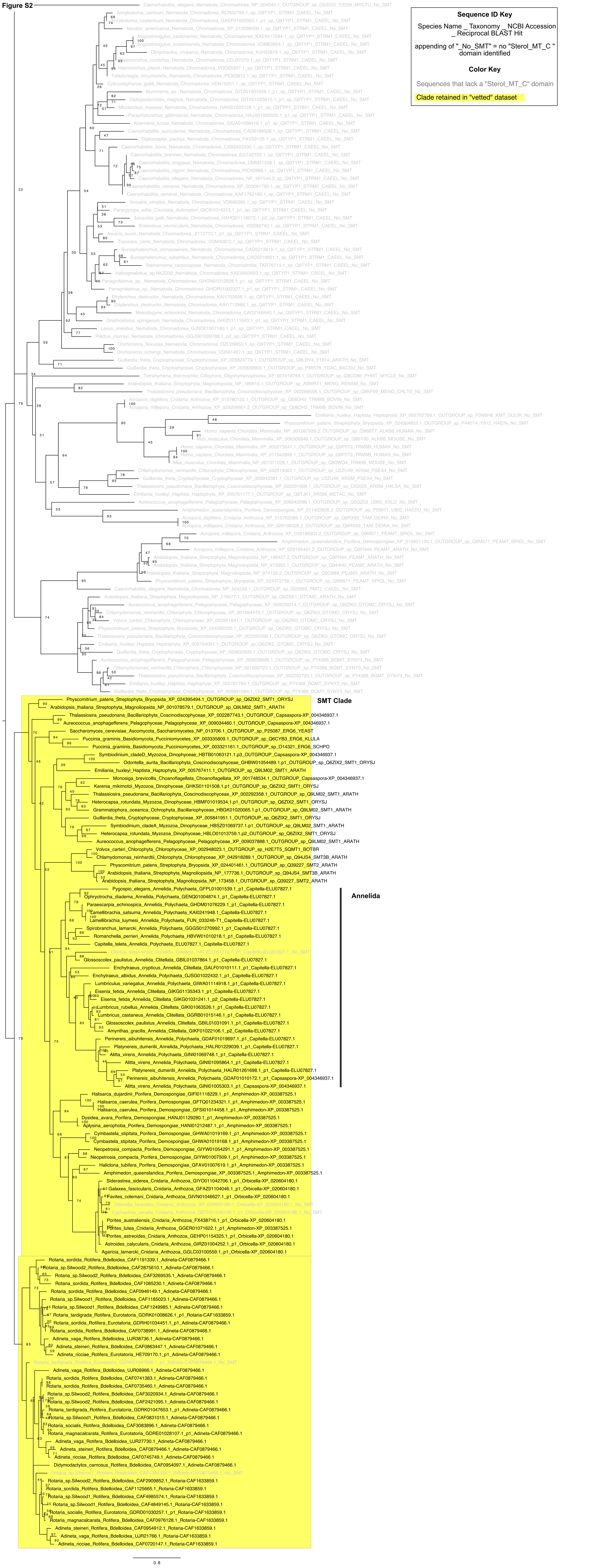
Sequence ID Key
Species Name _ Taxonomy _ NCBI Accession _ Reciprocal BLAST Hit
appending of "_No_SMT" = no "Sterol_MT_C " domain identified
Color Key
Sequences that lack a "Sterol_MT_C" domain
Clade retained in "vetted" dataset
SMT Clade
Annelida
0.8

Figure S3

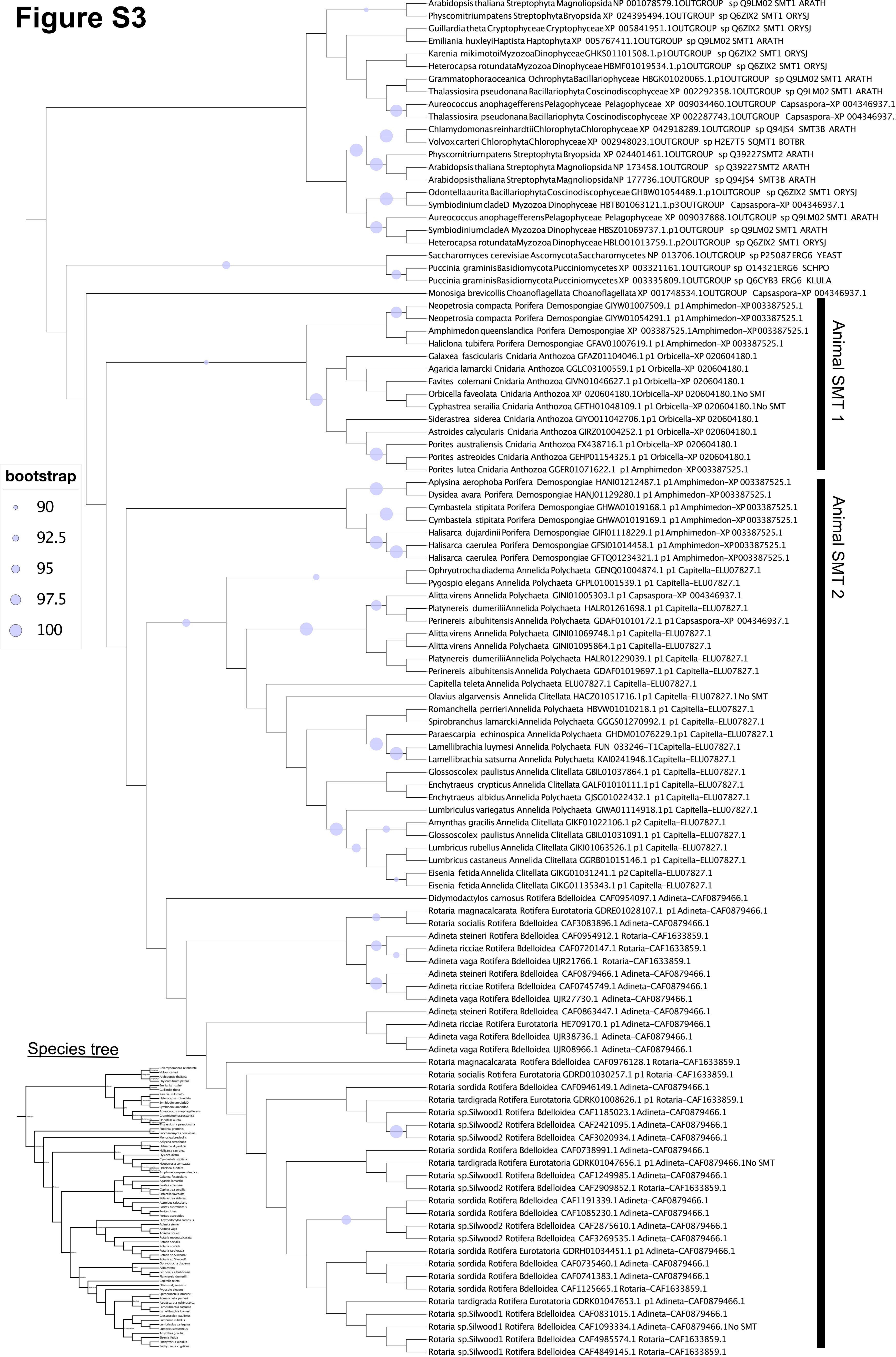
Arabidopsis thaliana Streptophyta Magnoliopsida NP 001078579.1OUTGROUP sp Q9LM02 SMT1 ARATH
Physcomitriumpatens Streptophyta Bryopsida XP 024395494.1OUTGROUP sp Q6ZIX2 SMT1 ORYSJ
Guillardia theta Cryptophyceae Cryptophyceae XP 005841951.1OUTGROUP sp Q6ZIX2 SMT1 ORYSJ
Emiliania huxleyi Haptista Haptophyta XP 005767411.1OUTGROUP sp Q9LM02 SMT1 ARATH
Karenia mikimotoi Myzozoa Dinophyceae GHKS01101508.1.p1OUTGROUP sp Q6ZIX2 SMT1 ORYSJ
Heterocapsa rotundata Myzozoa Dinophyceae HBMF01019534.1.p1OUTGROUP sp Q6ZIX2 SMT1 ORYSJ
Grammatophoraoceanica Ochrophyta Bacillariophyceae HBGK01020065.1.p1OUTGROUP sp Q9LM02 SMT1 ARATH
Thalassiosira pseudonana Bacillariophyta Coscinodiscophyceae XP 002292358.1OUTGROUP sp Q9LM02 SMT1 ARATH
Aureococcus anophagefferens Pelagophyceae Pelagophyceae XP 009034460.1OUTGROUP Capsaspora-XP 004346937.1
Thalassiosira pseudonana Bacillariophyta Coscinodiscophyceae XP 002287743.1OUTGROUP Capsaspora-XP 004346937.
Chlamydomonas reinhardtii Chlorophyta Chlorophyceae XP 042918289.1OUTGROUP sp Q94JS4 SMT3B ARATH
Volvox carteri Chlorophyta Chlorophyceae XP 002948023.1OUTGROUP sp H2E7T5 SQMT1 BOTBR
Physcomitrium patens Streptophyta Bryopsida XP 024401461.1OUTGROUP sp Q39227 SMT2 ARATH
Arabidopsis thaliana Streptophyta Magnoliopsida NP 173458.1OUTGROUP sp Q39227 SMT2 ARATH
Arabidopsis thaliana Streptophyta Magnoliopsida NP 177736.1OUTGROUP sp Q94JS4 SMT3B ARATH
Odontella aurita Bacillariophyta Coscinodiscophyceae GHBW01054489.1.p1OUTGROUP sp Q6ZIX2 SMT1 ORYSJ
Symbiodinium cladeD Myzozoa Dinophyceae HBTB01063121.1.p3OUTGROUP Capsaspora-XP 004346937.1
Aureococcus anophagefferens Pelagophyceae Pelagophyceae XP 009037888.1OUTGROUP sp Q9LM02 SMT1 ARATH
Symbiodinium cladeA Myzozoa Dinophyceae HBSZ01069737.1.p1OUTGROUP sp Q9LM02 SMT1 ARATH
Heterocapsa rotundata Myzozoa Dinophyceae HBLO01013759.1.p2OUTGROUP sp Q6ZIX2 SMT1 ORYSJ
Saccharomyces cerevisiae Ascomycota Saccharomycetes NP 013706.1OUTGROUP sp P25087 ERG6 YEAST
Puccinia graminis Basidiomycota Pucciniomycetes XP 003321161.1OUTGROUP sp O14321 ERG6 SCHPO
Puccinia graminis Basidiomycota Pucciniomycetes XP 003335809.1OUTGROUP sp Q6CYB3 ERG6 KLULA
Monosiga brevicollis Choanoflagellata Choanoflagellata XP 001748534.1OUTGROUP Capsaspora-XP 004346937.1
Neopetrosia compacta Porifera Demospongiae GIYW01007509.1 p1 Amphimedon-XP 003387525.1
Neopetrosia compacta Porifera Demospongiae GIYW01054291.1 p1 Amphimedon-XP 003387525.1
Amphimedon queenslandica Porifera Demospongiae XP 003387525.1Amphimedon-XP 003387525.1
Haliclona tubifera Porifera Demospongiae GFAV01007619.1 p1 Amphimedon-XP 003387525.1
Galaxea fascicularis Cnidaria Anthozoa GFAZ01104046.1 p1 Orbicella-XP 020604180.1
Agaricia lamarcki Cnidaria Anthozoa GGLC03100559.1 p1 Orbicella-XP 020604180.1
Favites colemani Cnidaria Anthozoa GIVN01046627.1 p1 Orbicella-XP 020604180.1
Orbicella faveolata Cnidaria Anthozoa XP 020604180.1Orbicella-XP 020604180.1No SMT
Cyphastrea serailia Cnidaria Anthozoa GETH01048109.1 p1 Orbicella-XP 020604180.1No SMT
Siderastrea siderea Cnidaria Anthozoa GIYO011042706.1 p1 Orbicella-XP 020604180.1
Astroides calycularis Cnidaria Anthozoa GIRZ01004252.1 p1 Orbicella-XP 020604180.1
Porites australiensis Cnidaria Anthozoa FX438716.1 p1 Orbicella-XP 020604180.1
Porites astreoides Cnidaria Anthozoa GEHP01154325.1 p1 Orbicella-XP 020604180.1
Porites lutea Cnidaria Anthozoa GGER01071622.1 p1 Amphimedon-XP 003387525.1
Aplysina aerophoba Porifera Demospongiae HANI01212487.1 p1 Amphimedon-XP 003387525.1
Dysidea avara Porifera Demospongiae HANJ01129280.1 p1 Amphimedon-XP 003387525.1
Cymbastela stipitata Porifera Demospongiae GHWA01019168.1 p1 Amphimedon-XP 003387525.1
Cymbastela stipitata Porifera Demospongiae GHWA01019169.1 p1 Amphimedon-XP 003387525.1
Halisarca dujardinii Porifera Demospongiae GIFI01118229.1 p1 Amphimedon-XP 003387525.1
Halisarca caerulea Porifera Demospongiae GFSI01014458.1 p1 Amphimedon-XP 003387525.1
Halisarca caerulea Porifera Demospongiae GFTQ01234321.1 p1 Amphimedon-XP 003387525.1
Ophryotrocha diadema Annelida Polychaeta GENQ01004874.1 p1 Capitella-ELU07827.1
Pygospio elegans Annelida Polychaeta GFPL01001539.1 p1 Capitella-ELU07827.1
Alitta virens Annelida Polychaeta GINI01005303.1 p1 Capsaspora-XP 004346937.1
Platynereis dumerilii Annelida Polychaeta HALR01261698.1 p1 Capitella-ELU07827.1
Perinereis aibuhitensis Annelida Polychaeta GDAF01010172.1 p1 Capsaspora-XP 004346937.1
Alitta virens Annelida Polychaeta GINI01069748.1 p1 Capitella-ELU07827.1
Alitta virens Annelida Polychaeta GINI01095864.1 p1 Capitella-ELU07827.1
Platynereis dumerilii Annelida Polychaeta HALR01229039.1 p1 Capitella-ELU07827.1
Perinereis aibuhitensis Annelida Polychaeta GDAF01019697.1 p1 Capitella-ELU07827.1
Capitella teleta Annelida Polychaeta ELU07827.1 Capitella-ELU07827.1
Olavius algarvensis Annelida Clitellata HACZ01051716.1 p1 Capitella-ELU07827.1 No SMT
Romanchella perrieri Annelida Polychaeta HBVW01010218.1 p1 Capitella-ELU07827.1
Spirobranchus lamarcki Annelida Polychaeta GGGS01270992.1 p1 Capitella-ELU07827.1
Paraescarpia echinospica Annelida Polychaeta GHDM01076229.1 p1 Capitella-ELU07827.1
Lamellibrachia luymesi Annelida Polychaeta FUN 033246-T1 Capitella-ELU07827.1
Lamellibrachia satsuma Annelida Polychaeta KAI0241948.1 Capitella-ELU07827.1
Glossoscolex paulistus Annelida Clitellata GBIL01037864.1 p1 Capitella-ELU07827.1
Enchytraeus crypticus Annelida Clitellata GALF01010111.1 p1 Capitella-ELU07827.1
Enchytraeus albidus Annelida Polychaeta GJSG01022432.1 p1 Capitella-ELU07827.1
Lumbriculus variegatus Annelida Polychaeta GIWA01114918.1 p1 Capitella-ELU07827.1
Amynthas gracilis Annelida Clitellata GIKF01022106.1 p2 Capitella-ELU07827.1
Glossoscolex paulistus Annelida Clitellata GBIL01031091.1 p1 Capitella-ELU07827.1
Lumbricus rubellus Annelida Clitellata GIKI01063526.1 p1 Capitella-ELU07827.1
Lumbricus castaneus Annelida Clitellata GGRB01015146.1 p1 Capitella-ELU07827.1
Eisenia fetida Annelida Clitellata GIKG01031241.1 p2 Capitella-ELU07827.1
Eisenia fetida Annelida Clitellata GIKG01135343.1 p1 Capitella-ELU07827.1
Didymodactylos carnosus Rotifera Bdelloidea CAF0954097.1 Adineta-CAF0879466.1
Rotaria magnacalcarata Rotifera Eurotatoria GDRE01028107.1 p1 Adineta-CAF0879466.1
Rotaria socialis Rotifera Bdelloidea CAF3083896.1 Adineta-CAF0879466.1
Adineta steineri Rotifera Bdelloidea CAF0954912.1 Rotaria-CAF1633859.1
Adineta ricciae Rotifera Bdelloidea CAF0720147.1 Rotaria-CAF1633859.1
Adineta vaga Rotifera Bdelloidea UJR21766.1 Rotaria-CAF1633859.1
Adineta steineri Rotifera Bdelloidea CAF0879466.1 Adineta-CAF0879466.1
Adineta ricciae Rotifera Bdelloidea CAF0745749.1 Adineta-CAF0879466.1
Adineta vaga Rotifera Bdelloidea UJR27730.1 Adineta-CAF0879466.1
Adineta steineri Rotifera Bdelloidea CAF0863447.1 Adineta-CAF0879466.1
Adineta ricciae Rotifera Eurotatoria HE709170.1 p1 Adineta-CAF0879466.1
Adineta vaga Rotifera Bdelloidea UJR38736.1 Adineta-CAF0879466.1
Adineta vaga Rotifera Bdelloidea UJR08966.1 Adineta-CAF0879466.1
Rotaria magnacalcarata Rotifera Bdelloidea CAF0976128.1 Rotaria-CAF1633859.1
Rotaria socialis Rotifera Eurotatoria GDRD01030257.1 p1 Rotaria-CAF1633859.1
Rotaria sordida Rotifera Bdelloidea CAF0946149.1 Adineta-CAF0879466.1
Rotaria tardigrada Rotifera Eurotatoria GDRK01008626.1 p1 Rotaria-CAF1633859.1
Rotaria sp.Silwood1 Rotifera Bdelloidea CAF1185023.1 Adineta-CAF0879466.1
Rotaria sp.Silwood2 Rotifera Bdelloidea CAF2421095.1 Adineta-CAF0879466.1
Rotaria sp.Silwood2 Rotifera Bdelloidea CAF3020934.1 Adineta-CAF0879466.1
Rotaria sordida Rotifera Bdelloidea CAF0738991.1 Adineta-CAF0879466.1
Rotaria tardigrada Rotifera Eurotatoria GDRK01047656.1 p1 Adineta-CAF0879466.1No SMT
Rotaria sp.Silwood1 Rotifera Bdelloidea CAF1249985.1 Adineta-CAF0879466.1
Rotaria sp.Silwood2 Rotifera Bdelloidea CAF2909852.1 Rotaria-CAF1633859.1
Rotaria sordida Rotifera Bdelloidea CAF1191339.1 Adineta-CAF0879466.1
Rotaria sordida Rotifera Bdelloidea CAF1085230.1 Adineta-CAF0879466.1
Rotaria sp.Silwood2 Rotifera Bdelloidea CAF2875610.1 Adineta-CAF0879466.1
Rotaria sp.Silwood2 Rotifera Bdelloidea CAF3269535.1 Adineta-CAF0879466.1
Rotaria sordida Rotifera Eurotatoria GDRH01034451.1 p1 Adineta-CAF0879466.1
Rotaria sordida Rotifera Bdelloidea CAF0735460.1 Adineta-CAF0879466.1
Rotaria sordida Rotifera Bdelloidea CAF0741383.1 Adineta-CAF0879466.1
Rotaria sordida Rotifera Bdelloidea CAF1125665.1 Rotaria-CAF1633859.1
Rotaria tardigrada Rotifera Eurotatoria GDRK01047653.1 p1 Adineta-CAF0879466.1
Rotaria sp.Silwood1 Rotifera Bdelloidea CAF0831015.1 Adineta-CAF0879466.1
Rotaria sp.Silwood1 Rotifera Bdelloidea CAF1093334.1 Adineta-CAF0879466.1No SMT
Rotaria sp.Silwood1 Rotifera Bdelloidea CAF4985574.1 Rotaria-CAF1633859.1
Rotaria sp.Silwood1 Rotifera Bdelloidea CAF4849145.1 Rotaria-CAF1633859.1
Animal SMT 1
Animal SMT 2
bootstrap
90
92.5
95
97.5
100
Species tree

Figure S4

Porites australiensis genome (GCA_022179025.1): contig BOPM01000048.1

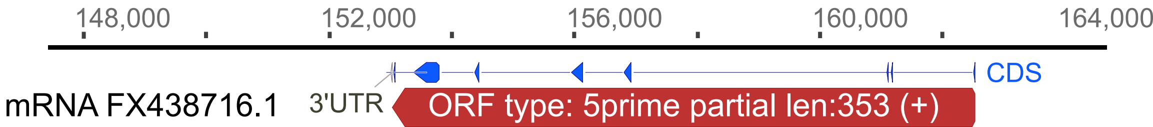

Figure S5

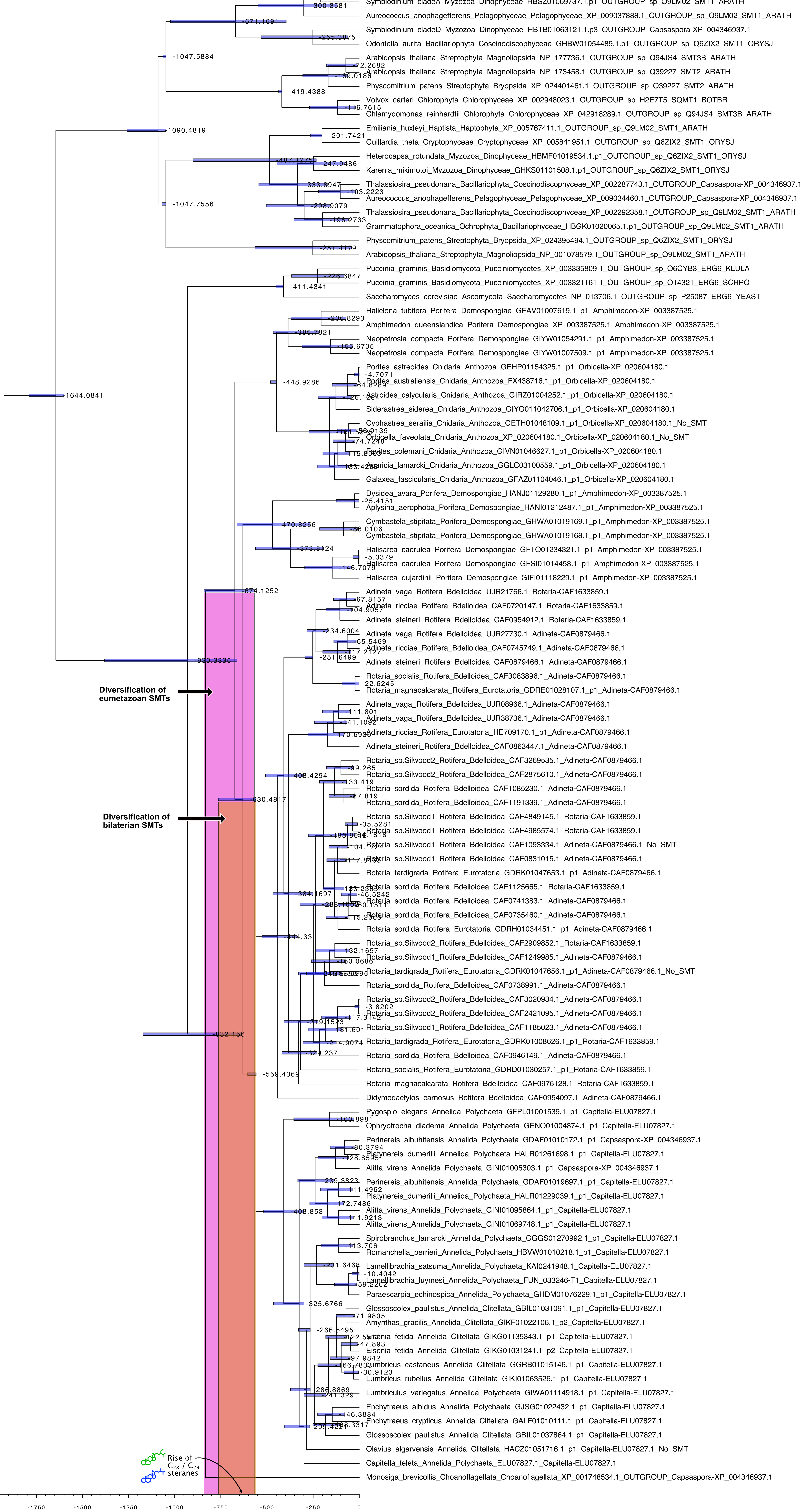

Heterocapsa_rotundata_Myzozoa_Dinophyceae_HBLO01013759.1.p2_OUTGROUP_sp_Q6ZIX2_SMT1_ORYSJ
Symbiodinium_cladeA_Myzozoa_Dinophyceae_HBSZ01069737.1.p1_OUTGROUP_sp_Q9LM02_SMT1_ARATH
Aureococcus_anophagefferens_Pelagophyceae_Pelagophyceae_XP_009037888.1_OUTGROUP_sp_Q9LM02_SMT1_ARATH
Symbiodinium_cladeD_Myzozoa_Dinophyceae_HBTB01063121.1.p3_OUTGROUP_Capsaspora-XP_004346937.1
Odontella_aurita_Bacillariophyta_Coscinodiscophyceae_GHBW01054489.1.p1_OUTGROUP_sp_Q6ZIX2_SMT1_ORYSJ
Arabidopsis_thaliana_Streptophyta_Magnoliopsida_NP_177736.1_OUTGROUP_sp_Q94JS4_SMT3B_ARATH
Arabidopsis_thaliana_Streptophyta_Magnoliopsida_NP_173458.1_OUTGROUP_sp_Q39227_SMT2_ARATH
Physcomitrium_patens_Streptophyta_Bryopsida_XP_024401461.1_OUTGROUP_sp_Q39227_SMT2_ARATH
Volvox_carteri_Chlorophyta_Chlorophyceae_XP_002948023.1_OUTGROUP_sp_H2E7T5_SQMT1_BOTBR
Chlamydomonas_reinhardtii_Chlorophyta_Chlorophyceae_XP_042918289.1_OUTGROUP_sp_Q94JS4_SMT3B_ARATH
Emiliania_huxleyi_Haptista_Haptophyta_XP_005767411.1_OUTGROUP_sp_Q9LM02_SMT1_ARATH
Guillardia_theta_Cryptophyceae_Cryptophyceae_XP_005841951.1_OUTGROUP_sp_Q6ZIX2_SMT1_ORYSJ
Heterocapsa_rotundata_Myzozoa_Dinophyceae_HBMF01019534.1.p1_OUTGROUP_sp_Q6ZIX2_SMT1_ORYSJ
Karenia_mikimotoi_Myzozoa_Dinophyceae_GHKS01101508.1.p1_OUTGROUP_sp_Q6ZIX2_SMT1_ORYSJ
Thalassiosira_pseudonana_Bacillariophyta_Coscinodiscophyceae_XP_002287743.1_OUTGROUP_Capsaspora-XP_004346937.1
Aureococcus_anophagefferens_Pelagophyceae_Pelagophyceae_XP_009034460.1_OUTGROUP_Capsaspora-XP_004346937.1
Thalassiosira_pseudonana_Bacillariophyta_Coscinodiscophyceae_XP_002292358.1_OUTGROUP_sp_Q9LM02_SMT1_ARATH
Grammatophora_oceanica_Ochrophyta_Bacillariophyceae_HBGK01020065.1.p1_OUTGROUP_sp_Q9LM02_SMT1_ARATH
Physcomitrium_patens_Streptophyta_Bryopsida_XP_024395494.1_OUTGROUP_sp_Q6ZIX2_SMT1_ORYSJ
Arabidopsis_thaliana_Streptophyta_Magnoliopsida_NP_001078579.1_OUTGROUP_sp_Q9LM02_SMT1_ARATH
Puccinia_graminis_Basidiomycota_Pucciniomycetes_XP_003335809.1_OUTGROUP_sp_Q6CYB3_ERG6_KLULA
Puccinia_graminis_Basidiomycota_Pucciniomycetes_XP_003321161.1_OUTGROUP_sp_O14321_ERG6_SCHPO
Saccharomyces_cerevisiae_Ascomycota_Saccharomycetes_NP_013706.1_OUTGROUP_sp_P25087_ERG6_YEAST
Haliclona_tubifera_Porifera_Demospongiae_GFAV01007619.1_p1_Amphimedon-XP_003387525.1
Amphimedon_queenslandica_Porifera_Demospongiae_XP_003387525.1_Amphimedon-XP_003387525.1
Neopetrosia_compacta_Porifera_Demospongiae_GIYW01054291.1_p1_Amphimedon-XP_003387525.1
Neopetrosia_compacta_Porifera_Demospongiae_GIYW01007509.1_p1_Amphimedon-XP_003387525.1
Porites_astreoides_Cnidaria_Anthozoa_GEHP01154325.1_p1_Orbicella-XP_020604180.1
Porites_australiensis_Cnidaria_Anthozoa_FX438716.1_p1_Orbicella-XP_020604180.1
Astroides_calycularis_Cnidaria_Anthozoa_GIRZ01004252.1_p1_Orbicella-XP_020604180.1
Siderastrea_siderea_Cnidaria_Anthozoa_GIYO011042706.1_p1_Orbicella-XP_020604180.1
Cyphastrea_serailia_Cnidaria_Anthozoa_GETH01048109.1_p1_Orbicella-XP_020604180.1_No_SMT
Orbicella_faveolata_Cnidaria_Anthozoa_XP_020604180.1_Orbicella-XP_020604180.1_No_SMT
Favites_colemani_Cnidaria_Anthozoa_GIVN01046627.1_p1_Orbicella-XP_020604180.1
Agaricia_lamarcki_Cnidaria_Anthozoa_GGLC03100559.1_p1_Orbicella-XP_020604180.1
Galaxea_fascicularis_Cnidaria_Anthozoa_GFAZ01104046.1_p1_Orbicella-XP_020604180.1
Dysidea_avara_Porifera_Demospongiae_HANJ01129280.1_p1_Amphimedon-XP_003387525.1
Aplysina_aerophoba_Porifera_Demospongiae_HANI01212487.1_p1_Amphimedon-XP_003387525.1
Cymbastela_stipitata_Porifera_Demospongiae_GHWA01019169.1_p1_Amphimedon-XP_003387525.1
Cymbastela_stipitata_Porifera_Demospongiae_GHWA01019168.1_p1_Amphimedon-XP_003387525.1
Halisarca_caerulea_Porifera_Demospongiae_GFTQ01234321.1_p1_Amphimedon-XP_003387525.1
Halisarca_caerulea_Porifera_Demospongiae_GFSI01014458.1_p1_Amphimedon-XP_003387525.1
Halisarca_dujardinii_Porifera_Demospongiae_GIFI01118229.1_p1_Amphimedon-XP_003387525.1
Adineta_vaga_Rotifera_Bdelloidea_UJR21766.1_Rotaria-CAF1633859.1
Adineta_ricciae_Rotifera_Bdelloidea_CAF0720147.1_Rotaria-CAF1633859.1
Adineta_steineri_Rotifera_Bdelloidea_CAF0954912.1_Rotaria-CAF1633859.1
Adineta_vaga_Rotifera_Bdelloidea_UJR27730.1_Adineta-CAF0879466.1
Adineta_ricciae_Rotifera_Bdelloidea_CAF0745749.1_Adineta-CAF0879466.1
Adineta_steineri_Rotifera_Bdelloidea_CAF0879466.1_Adineta-CAF0879466.1
Rotaria_socialis_Rotifera_Bdelloidea_CAF3083896.1_Adineta-CAF0879466.1
Rotaria_magnacalcarata_Rotifera_Eurotatoria_GDRE01028107.1_p1_Adineta-CAF0879466.1
Adineta_vaga_Rotifera_Bdelloidea_UJR08966.1_Adineta-CAF0879466.1
Adineta_vaga_Rotifera_Bdelloidea_UJR38736.1_Adineta-CAF0879466.1
Adineta_ricciae_Rotifera_Eurotatoria_HE709170.1_p1_Adineta-CAF0879466.1
Adineta_steineri_Rotifera_Bdelloidea_CAF0863447.1_Adineta-CAF0879466.1
Rotaria_sp.Silwood2_Rotifera_Bdelloidea_CAF3269535.1_Adineta-CAF0879466.1
Rotaria_sp.Silwood2_Rotifera_Bdelloidea_CAF2875610.1_Adineta-CAF0879466.1
Rotaria_sordida_Rotifera_Bdelloidea_CAF1085230.1_Adineta-CAF0879466.1
Rotaria_sordida_Rotifera_Bdelloidea_CAF1191339.1_Adineta-CAF0879466.1
Rotaria_sp.Silwood1_Rotifera_Bdelloidea_CAF4849145.1_Rotaria-CAF1633859.1
Rotaria_sp.Silwood1_Rotifera_Bdelloidea_CAF4985574.1_Rotaria-CAF1633859.1
Rotaria_sp.Silwood1_Rotifera_Bdelloidea_CAF1093334.1_Adineta-CAF0879466.1_No_SMT
Rotaria_sp.Silwood1_Rotifera_Bdelloidea_CAF0831015.1_Adineta-CAF0879466.1
Rotaria_tardigrada_Rotifera_Eurotatoria_GDRK01047653.1_p1_Adineta-CAF0879466.1
Rotaria_sordida_Rotifera_Bdelloidea_CAF1125665.1_Rotaria-CAF1633859.1
Rotaria_sordida_Rotifera_Bdelloidea_CAF0741383.1_Adineta-CAF0879466.1
Rotaria_sordida_Rotifera_Bdelloidea_CAF0735460.1_Adineta-CAF0879466.1
Rotaria_sordida_Rotifera_Eurotatoria_GDRH01034451.1_p1_Adineta-CAF0879466.1
Rotaria_sp.Silwood2_Rotifera_Bdelloidea_CAF2909852.1_Rotaria-CAF1633859.1
Rotaria_sp.Silwood1_Rotifera_Bdelloidea_CAF1249985.1_Adineta-CAF0879466.1
Rotaria_tardigrada_Rotifera_Eurotatoria_GDRK01047656.1_p1_Adineta-CAF0879466.1_No_SMT
Rotaria_sordida_Rotifera_Bdelloidea_CAF0738991.1_Adineta-CAF0879466.1
Rotaria_sp.Silwood2_Rotifera_Bdelloidea_CAF3020934.1_Adineta-CAF0879466.1
Rotaria_sp.Silwood2_Rotifera_Bdelloidea_CAF2421095.1_Adineta-CAF0879466.1
Rotaria_sp.Silwood1_Rotifera_Bdelloidea_CAF1185023.1_Adineta-CAF0879466.1
Rotaria_tardigrada_Rotifera_Eurotatoria_GDRK01008626.1_p1_Rotaria-CAF1633859.1
Rotaria_sordida_Rotifera_Bdelloidea_CAF0946149.1_Adineta-CAF0879466.1
Rotaria_socialis_Rotifera_Eurotatoria_GDRD01030257.1_p1_Rotaria-CAF1633859.1
Rotaria_magnacalcarata_Rotifera_Bdelloidea_CAF0976128.1_Rotaria-CAF1633859.1
Didymodactylos_carnosus_Rotifera_Bdelloidea_CAF0954097.1_Adineta-CAF0879466.1
Pygospio_elegans_Annelida_Polychaeta_GFPL01001539.1_p1_Capitella-ELU07827.1
Ophryotrocha_diadema_Annelida_Polychaeta_GENQ01004874.1_p1_Capitella-ELU07827.1
Perinereis_aibuhitensis_Annelida_Polychaeta_GDAF01010172.1_p1_Capsaspora-XP_004346937.1
Platynereis_dumerilii_Annelida_Polychaeta_HALR01261698.1_p1_Capitella-ELU07827.1
Alitta_virens_Annelida_Polychaeta_GINI01005303.1_p1_Capsaspora-XP_004346937.1
Perinereis_aibuhitensis_Annelida_Polychaeta_GDAF01019697.1_p1_Capitella-ELU07827.1
Platynereis_dumerilii_Annelida_Polychaeta_HALR01229039.1_p1_Capitella-ELU07827.1
Alitta_virens_Annelida_Polychaeta_GINI01095864.1_p1_Capitella-ELU07827.1
Alitta_virens_Annelida_Polychaeta_GINI01069748.1_p1_Capitella-ELU07827.1
Spirobranchus_lamarcki_Annelida_Polychaeta_GGGS01270992.1_p1_Capitella-ELU07827.1
Romanchella_perrieri_Annelida_Polychaeta_HBVW01010218.1_p1_Capitella-ELU07827.1
Lamellibrachia_satsuma_Annelida_Polychaeta_KAI0241948.1_Capitella-ELU07827.1
Lamellibrachia_luymesi_Annelida_Polychaeta_FUN_033246-T1_Capitella-ELU07827.1
Paraescarpia_echinospica_Annelida_Polychaeta_GHDM01076229.1_p1_Capitella-ELU07827.1
Glossoscolex_paulistus_Annelida_Clitellata_GBIL01031091.1_p1_Capitella-ELU07827.1
Amynthas_gracilis_Annelida_Clitellata_GIKF01022106.1_p2_Capitella-ELU07827.1
Eisenia_fetida_Annelida_Clitellata_GIKG01135343.1_p1_Capitella-ELU07827.1
Eisenia_fetida_Annelida_Clitellata_GIKG01031241.1_p2_Capitella-ELU07827.1
Lumbricus_castaneus_Annelida_Clitellata_GGRB01015146.1_p1_Capitella-ELU07827.1
Lumbricus_rubellus_Annelida_Clitellata_GIKI01063526.1_p1_Capitella-ELU07827.1
Lumbriculus_variegatus_Annelida_Polychaeta_GIWA01114918.1_p1_Capitella-ELU07827.1
Enchytraeus_albidus_Annelida_Polychaeta_GJSG01022432.1_p1_Capitella-ELU07827.1
Enchytraeus_crypticus_Annelida_Clitellata_GALF01010111.1_p1_Capitella-ELU07827.1
Glossoscolex_paulistus_Annelida_Clitellata_GBIL01037864.1_p1_Capitella-ELU07827.1
Olavius_algarvensis_Annelida_Clitellata_HACZ01051716.1_p1_Capitella-ELU07827.1_No_SMT
Capitella_teleta_Annelida_Polychaeta_ELU07827.1_Capitella-ELU07827.1
Monosiga_brevicollis_Choanoflagellata_Choanoflagellata_XP_001748534.1_OUTGROUP_Capsaspora-XP_004346937.1
Diversification of eumetazoan SMTs
Diversification of bilaterian SMTs
Rise of C_{28} / C_{29} steranes
-1750
-1500
-1250
-1000
-750
-500
-250
0

Figure S6

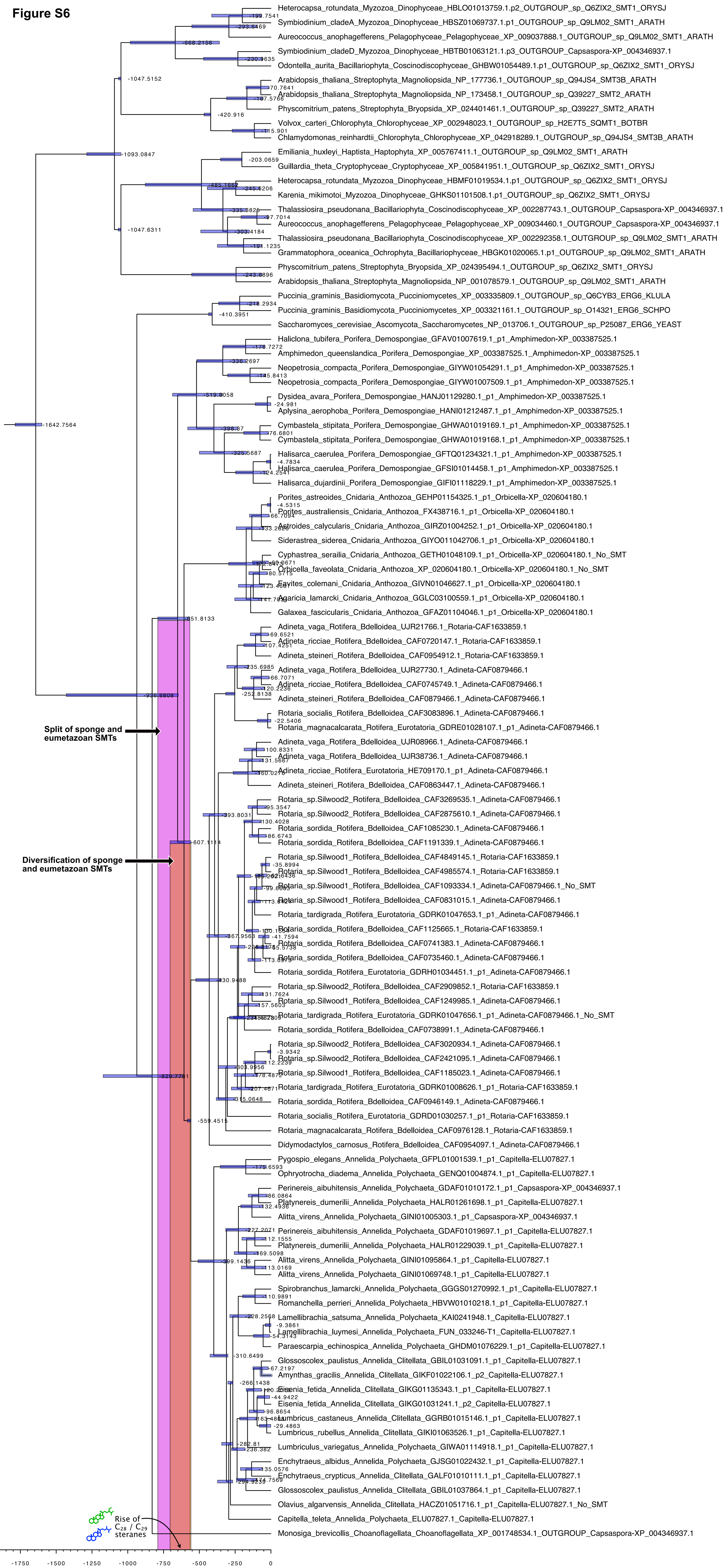

Heterocapsa_rotundata_Myzozoa_Dinophyceae_HBLO01013759.1.p2_OUTGROUP_sp_Q6ZIX2_SMT1_ORYSJ
Symbiodinium_cladeA_Myzozoa_Dinophyceae_HBSZ01069737.1.p1_OUTGROUP_sp_Q9LM02_SMT1_ARATH
Aureococcus_anophagefferens_Pelagophyceae_Pelagophyceae_XP_009037888.1_OUTGROUP_sp_Q9LM02_SMT1_ARATH
Symbiodinium_cladeD_Myzozoa_Dinophyceae_HBTB01063121.1.p3_OUTGROUP_Capsaspora-XP_004346937.1
Odontella_aurita_Bacillariophyta_Coscinodiscophyceae_GHBW01054489.1.p1_OUTGROUP_sp_Q6ZIX2_SMT1_ORYSJ
Arabidopsis_thaliana_Streptophyta_Magnoliopsida_NP_177736.1_OUTGROUP_sp_Q94JS4_SMT3B_ARATH
Arabidopsis_thaliana_Streptophyta_Magnoliopsida_NP_173458.1_OUTGROUP_sp_Q39227_SMT2_ARATH
Physcomitrium_patens_Streptophyta_Bryopsida_XP_024401461.1_OUTGROUP_sp_Q39227_SMT2_ARATH
Volvox_carteri_Chlorophyta_Chlorophyceae_XP_002948023.1_OUTGROUP_sp_H2E7T5_SQMT1_BOTBR
Chlamydomonas_reinhardtii_Chlorophyta_Chlorophyceae_XP_042918289.1_OUTGROUP_sp_Q94JS4_SMT3B_ARATH
Emiliania_huxleyi_Haptista_Haptophyta_XP_005767411.1_OUTGROUP_sp_Q9LM02_SMT1_ARATH
Guillardia_theta_Cryptophyceae_Cryptophyceae_XP_005841951.1_OUTGROUP_sp_Q6ZIX2_SMT1_ORYSJ
Heterocapsa_rotundata_Myzozoa_Dinophyceae_HBMF01019534.1.p1_OUTGROUP_sp_Q6ZIX2_SMT1_ORYSJ
Karenia_mikimotoi_Myzozoa_Dinophyceae_GHKS01101508.1.p1_OUTGROUP_sp_Q6ZIX2_SMT1_ORYSJ
Thalassiosira_pseudonana_Bacillariophyta_Coscinodiscophyceae_XP_002287743.1_OUTGROUP_Capsaspora-XP_004346937.1
Aureococcus_anophagefferens_Pelagophyceae_Pelagophyceae_XP_009034460.1_OUTGROUP_Capsaspora-XP_004346937.1
Thalassiosira_pseudonana_Bacillariophyta_Coscinodiscophyceae_XP_002292358.1_OUTGROUP_sp_Q9LM02_SMT1_ARATH
Grammatophora_oceanica_Ochrophyta_Bacillariophyceae_HBGK01020065.1.p1_OUTGROUP_sp_Q9LM02_SMT1_ARATH
Physcomitrium_patens_Streptophyta_Bryopsida_XP_024395494.1_OUTGROUP_sp_Q6ZIX2_SMT1_ORYSJ
Arabidopsis_thaliana_Streptophyta_Magnoliopsida_NP_001078579.1_OUTGROUP_sp_Q9LM02_SMT1_ARATH
Puccinia_graminis_Basidiomycota_Pucciniomycetes_XP_003335809.1_OUTGROUP_sp_Q6CYB3_ERG6_KLULA
Puccinia_graminis_Basidiomycota_Pucciniomycetes_XP_003321161.1_OUTGROUP_sp_O14321_ERG6_SCHPO
Saccharomyces_cerevisiae_Ascomycota_Saccharomycetes_NP_013706.1_OUTGROUP_sp_P25087_ERG6_YEAST
Haliclona_tubifera_Porifera_Demospongiae_GFAV01007619.1_p1_Amphimedon-XP_003387525.1
Amphimedon_queenslandica_Porifera_Demospongiae_XP_003387525.1_Amphimedon-XP_003387525.1
Neopetrosia_compacta_Porifera_Demospongiae_GIYW01054291.1_p1_Amphimedon-XP_003387525.1
Neopetrosia_compacta_Porifera_Demospongiae_GIYW01007509.1_p1_Amphimedon-XP_003387525.1
Dysidea_avara_Porifera_Demospongiae_HANJ01129280.1_p1_Amphimedon-XP_003387525.1
Aplysina_aerophoba_Porifera_Demospongiae_HANI01212487.1_p1_Amphimedon-XP_003387525.1
Cymbastela_stipitata_Porifera_Demospongiae_GHWA01019169.1_p1_Amphimedon-XP_003387525.1
Cymbastela_stipitata_Porifera_Demospongiae_GHWA01019168.1_p1_Amphimedon-XP_003387525.1
Halisarca_caerulea_Porifera_Demospongiae_GFTQ01234321.1_p1_Amphimedon-XP_003387525.1
Halisarca_caerulea_Porifera_Demospongiae_GFSI01014458.1_p1_Amphimedon-XP_003387525.1
Halisarca_dujardinii_Porifera_Demospongiae_GIFI01118229.1_p1_Amphimedon-XP_003387525.1
Porites_astreoides_Cnidaria_Anthozoa_GEHP01154325.1_p1_Orbicella-XP_020604180.1
Porites_australiensis_Cnidaria_Anthozoa_FX438716.1_p1_Orbicella-XP_020604180.1
Astroides_calycularis_Cnidaria_Anthozoa_GIRZ01004252.1_p1_Orbicella-XP_020604180.1
Siderastrea_siderea_Cnidaria_Anthozoa_GIYO011042706.1_p1_Orbicella-XP_020604180.1
Cyphastrea_serailia_Cnidaria_Anthozoa_GETH01048109.1_p1_Orbicella-XP_020604180.1_No_SMT
Orbicella_faveolata_Cnidaria_Anthozoa_XP_020604180.1_Orbicella-XP_020604180.1_No_SMT
Favites_colemani_Cnidaria_Anthozoa_GIVN01046627.1_p1_Orbicella-XP_020604180.1
Agaricia_lamarcki_Cnidaria_Anthozoa_GGLC03100559.1_p1_Orbicella-XP_020604180.1
Galaxea_fascicularis_Cnidaria_Anthozoa_GFAZ01104046.1_p1_Orbicella-XP_020604180.1
Adineta_vaga_Rotifera_Bdelloidea_UJR21766.1_Rotaria-CAF1633859.1
Adineta_ricciae_Rotifera_Bdelloidea_CAF0720147.1_Rotaria-CAF1633859.1
Adineta_steineri_Rotifera_Bdelloidea_CAF0954912.1_Rotaria-CAF1633859.1
Adineta_vaga_Rotifera_Bdelloidea_UJR27730.1_Adineta-CAF0879466.1
Adineta_ricciae_Rotifera_Bdelloidea_CAF0745749.1_Adineta-CAF0879466.1
Adineta_steineri_Rotifera_Bdelloidea_CAF0879466.1_Adineta-CAF0879466.1
Rotaria_socialis_Rotifera_Bdelloidea_CAF3083896.1_Adineta-CAF0879466.1
Rotaria_magnacalcarata_Rotifera_Eurotatoria_GDRE01028107.1_p1_Adineta-CAF0879466.1
Adineta_vaga_Rotifera_Bdelloidea_UJR08966.1_Adineta-CAF0879466.1
Adineta_vaga_Rotifera_Bdelloidea_UJR38736.1_Adineta-CAF0879466.1
Adineta_ricciae_Rotifera_Eurotatoria_HE709170.1_p1_Adineta-CAF0879466.1
Adineta_steineri_Rotifera_Bdelloidea_CAF0863447.1_Adineta-CAF0879466.1
Rotaria_sp.Silwood2_Rotifera_Bdelloidea_CAF3269535.1_Adineta-CAF0879466.1
Rotaria_sp.Silwood2_Rotifera_Bdelloidea_CAF2875610.1_Adineta-CAF0879466.1
Rotaria_sordida_Rotifera_Bdelloidea_CAF1085230.1_Adineta-CAF0879466.1
Rotaria_sordida_Rotifera_Bdelloidea_CAF1191339.1_Adineta-CAF0879466.1
Rotaria_sp.Silwood1_Rotifera_Bdelloidea_CAF4849145.1_Rotaria-CAF1633859.1
Rotaria_sp.Silwood1_Rotifera_Bdelloidea_CAF4985574.1_Rotaria-CAF1633859.1
Rotaria_sp.Silwood1_Rotifera_Bdelloidea_CAF1093334.1_Adineta-CAF0879466.1_No_SMT
Rotaria_sp.Silwood1_Rotifera_Bdelloidea_CAF0831015.1_Adineta-CAF0879466.1
Rotaria_tardigrada_Rotifera_Eurotatoria_GDRK01047653.1_p1_Adineta-CAF0879466.1
Rotaria_sordida_Rotifera_Bdelloidea_CAF1125665.1_Rotaria-CAF1633859.1
Rotaria_sordida_Rotifera_Bdelloidea_CAF0741383.1_Adineta-CAF0879466.1
Rotaria_sordida_Rotifera_Bdelloidea_CAF0735460.1_Adineta-CAF0879466.1
Rotaria_sordida_Rotifera_Eurotatoria_GDRH01034451.1_p1_Adineta-CAF0879466.1
Rotaria_sp.Silwood2_Rotifera_Bdelloidea_CAF2909852.1_Rotaria-CAF1633859.1
Rotaria_sp.Silwood1_Rotifera_Bdelloidea_CAF1249985.1_Adineta-CAF0879466.1
Rotaria_tardigrada_Rotifera_Eurotatoria_GDRK01047656.1_p1_Adineta-CAF0879466.1_No_SMT
Rotaria_sordida_Rotifera_Bdelloidea_CAF0738991.1_Adineta-CAF0879466.1
Rotaria_sp.Silwood2_Rotifera_Bdelloidea_CAF3020934.1_Adineta-CAF0879466.1
Rotaria_sp.Silwood2_Rotifera_Bdelloidea_CAF2421095.1_Adineta-CAF0879466.1
Rotaria_sp.Silwood1_Rotifera_Bdelloidea_CAF1185023.1_Adineta-CAF0879466.1
Rotaria_tardigrada_Rotifera_Eurotatoria_GDRK01008626.1_p1_Rotaria-CAF1633859.1
Rotaria_sordida_Rotifera_Bdelloidea_CAF0946149.1_Adineta-CAF0879466.1
Rotaria_socialis_Rotifera_Eurotatoria_GDRD01030257.1_p1_Rotaria-CAF1633859.1
Rotaria_magnacalcarata_Rotifera_Bdelloidea_CAF0976128.1_Rotaria-CAF1633859.1
Didymodactylos_carnosus_Rotifera_Bdelloidea_CAF0954097.1_Adineta-CAF0879466.1
Pygospio_elegans_Annelida_Polychaeta_GFPL01001539.1_p1_Capitella-ELU07827.1
Ophryotrocha_diadema_Annelida_Polychaeta_GENQ01004874.1_p1_Capitella-ELU07827.1
Perinereis_aibuhitensis_Annelida_Polychaeta_GDAF01010172.1_p1_Capsaspora-XP_004346937.1
Platynereis_dumerilii_Annelida_Polychaeta_HALR01261698.1_p1_Capitella-ELU07827.1
Alitta_virens_Annelida_Polychaeta_GINI01005303.1_p1_Capsaspora-XP_004346937.1
Perinereis_aibuhitensis_Annelida_Polychaeta_GDAF01019697.1_p1_Capitella-ELU07827.1
Platynereis_dumerilii_Annelida_Polychaeta_HALR01229039.1_p1_Capitella-ELU07827.1
Alitta_virens_Annelida_Polychaeta_GINI01095864.1_p1_Capitella-ELU07827.1
Alitta_virens_Annelida_Polychaeta_GINI01069748.1_p1_Capitella-ELU07827.1
Spirobranchus_lamarcki_Annelida_Polychaeta_GGGS01270992.1_p1_Capitella-ELU07827.1
Romanchella_perrieri_Annelida_Polychaeta_HBVW01010218.1_p1_Capitella-ELU07827.1
Lamellibrachia_satsuma_Annelida_Polychaeta_KAI0241948.1_Capitella-ELU07827.1
Lamellibrachia_luymesi_Annelida_Polychaeta_FUN_033246-T1_Capitella-ELU07827.1
Paraescarpia_echinospica_Annelida_Polychaeta_GHDM01076229.1_p1_Capitella-ELU07827.1
Glossoscolex_paulistus_Annelida_Clitellata_GBIL01031091.1_p1_Capitella-ELU07827.1
Amynthas_gracilis_Annelida_Clitellata_GIKF01022106.1_p2_Capitella-ELU07827.1
Eisenia_fetida_Annelida_Clitellata_GIKG01135343.1_p1_Capitella-ELU07827.1
Eisenia_fetida_Annelida_Clitellata_GIKG01031241.1_p2_Capitella-ELU07827.1
Lumbricus_castaneus_Annelida_Clitellata_GGRB01015146.1_p1_Capitella-ELU07827.1
Lumbricus_rubellus_Annelida_Clitellata_GIKI01063526.1_p1_Capitella-ELU07827.1
Lumbriculus_variegatus_Annelida_Polychaeta_GIWA01114918.1_p1_Capitella-ELU07827.1
Enchytraeus_albidus_Annelida_Polychaeta_GJSG01022432.1_p1_Capitella-ELU07827.1
Enchytraeus_crypticus_Annelida_Clitellata_GALF01010111.1_p1_Capitella-ELU07827.1
Glossoscolex_paulistus_Annelida_Clitellata_GBIL01037864.1_p1_Capitella-ELU07827.1
Olavius_algarvensis_Annelida_Clitellata_HACZ01051716.1_p1_Capitella-ELU07827.1_No_SMT
Capitella_teleta_Annelida_Polychaeta_ELU07827.1_Capitella-ELU07827.1
Monosiga_brevicollis_Choanoflagellata_Choanoflagellata_XP_001748534.1_OUTGROUP_Capsaspora-XP_004346937.1
Split of sponge and eumetazoan SMTs
Diversification of sponge and eumetazoan SMTs
Rise of C_{28} / C_{29} steranes
-1750
-1500
-1250
-1000
-750
-500
-250
0

Figure S7

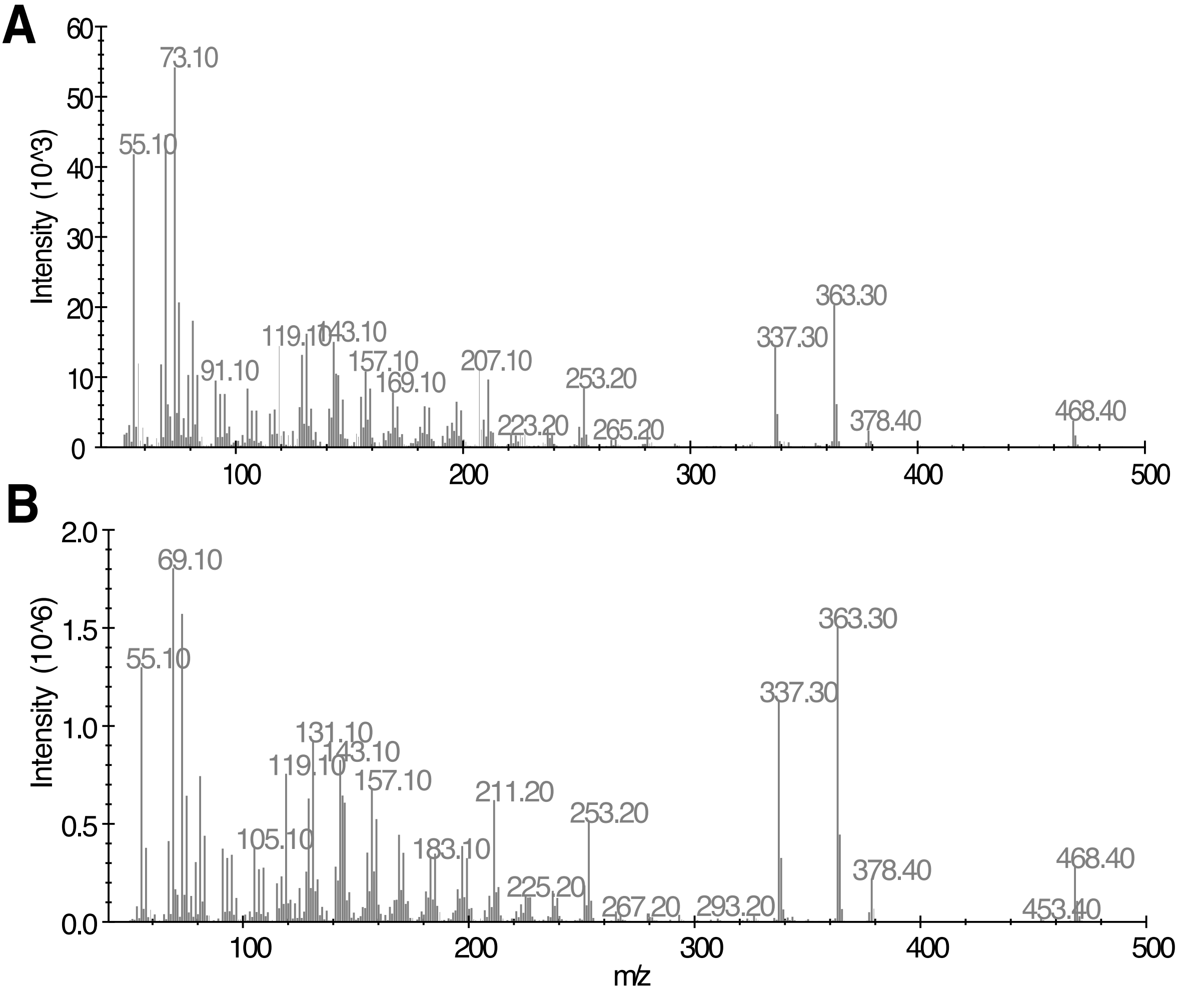

A
Intensity (10^3)
0
10
20
30
40
50
60
73.10
55.10
91.10
119.10
143.10
157.10
169.10
207.10
223.20
253.20
265.20
337.30
363.30
378.40
468.40
100
200
300
400
500
B
Intensity (10^6)
0.0
0.5
1.0
1.5
2.0
69.10
55.10
105.10
119.10
131.10
143.10
157.10
183.10
211.20
225.20
253.20
267.20
293.20
337.30
363.30
378.40
453.40
468.40
100
200
300
400
500
m/z
