# Supplementary material for: Common origin of sterol biosynthesis points to a feeding strategy shift in Neoproterozoic animals
Source: Nat Commun. 2023 Dec 1;14:7941. doi: 10.1038/s41467-023-43545-z (PMC10692144; doi:10.1038/s41467-023-43545-z)
Supplement: Supplementary file 5 — Reporting Summary [file 41467_2023_43545_MOESM5_ESM.pdf]

nature portfolio

Corresponding author(s): David Gold

Last updated by author(s): Nov 1, 2023

Reporting Summary

Nature Portfolio wishes to improve the reproducibility of the work that we publish. This form provides structure for consistency and transparency in reporting. For further information on Nature Portfolio policies, see our Editorial Policies and the Editorial Policy Checklist.

Statistics

For all statistical analyses, confirm that the following items are present in the figure legend, table legend, main text, or Methods section.

n/a	Confirmed	
☒	☐	The exact sample size (*n*) for each experimental group/condition, given as a discrete number and unit of measurement
☒	☐	A statement on whether measurements were taken from distinct samples or whether the same sample was measured repeatedly
☒	☐	The statistical test(s) used AND whether they are one- or two-sided<br>*Only common tests should be described solely by name; describe more complex techniques in the Methods section.*
☒	☐	A description of all covariates tested
☒	☐	A description of any assumptions or corrections, such as tests of normality and adjustment for multiple comparisons
☐	☒	A full description of the statistical parameters including central tendency (e.g. means) or other basic estimates (e.g. regression coefficient) AND variation (e.g. standard deviation) or associated estimates of uncertainty (e.g. confidence intervals)
☒	☐	For null hypothesis testing, the test statistic (e.g. *F*, *t*, *r*) with confidence intervals, effect sizes, degrees of freedom and *P* value noted<br>*Give P values as exact values whenever suitable.*
☒	☐	For Bayesian analysis, information on the choice of priors and Markov chain Monte Carlo settings
☒	☐	For hierarchical and complex designs, identification of the appropriate level for tests and full reporting of outcomes
☒	☐	Estimates of effect sizes (e.g. Cohen's *d*, Pearson's *r*), indicating how they were calculated

Our web collection on statistics for biologists contains articles on many of the points above.

Software and code

Policy information about availability of computer code

Data collection	The following open source tools were used for data collection: BLAST (v2.14.1+)
Data analysis	The following open source tools were used for data analysis: TransDecoder (v5.5.0); CD-HIT (v4.8.1); HMMER (v3.4); SAMTOOLS (v1.13); MAAFT (v7.490); trimAl (v1.4); IQTree (v1.6.12); Mesquite (v3.6.1); NOTUNG (v2.1.5); BEAST (v1.10.5); I-TASSER server; Pymol (v2.5.0); GMAP (v2019-09-12)

For manuscripts utilizing custom algorithms or software that are central to the research but not yet described in published literature, software must be made available to editors and reviewers. We strongly encourage code deposition in a community repository (e.g. GitHub). See the Nature Portfolio guidelines for submitting code & software for further information.

Data

Policy information about availability of data

All manuscripts must include a data availability statement. This statement should provide the following information, where applicable:

- Accession codes, unique identifiers, or web links for publicly available datasets
- A description of any restrictions on data availability
- For clinical datasets or third party data, please ensure that the statement adheres to our policy

All data used in this study, including scripts executed, input files, intermediate files, and output, are provided on GitHub at https://github.com/DavidGoldLab/2022_Annelid_SMTs. The accession numbers for all previously published genetic data can be found on GitHub in Supplementary Data file 1. The data generated in this study have been deposited in the Zenodo database under accession code https://zenodo.org/doi/10.5281/zenodo.10063989

Research involving human participants, their data, or biological material

Policy information about studies with human participants or human data. See also policy information about sex, gender (identity/presentation), and sexual orientation and race, ethnicity and racism.

Reporting on sex and gender	N/A
Reporting on race, ethnicity, or other socially relevant groupings	N/A
Population characteristics	N/A
Recruitment	N/A
Ethics oversight	N/A

Note that full information on the approval of the study protocol must also be provided in the manuscript.

Field-specific reporting

Please select the one below that is the best fit for your research. If you are not sure, read the appropriate sections before making your selection.

☐ Life sciences ☐ Behavioural & social sciences ☒ Ecological, evolutionary & environmental sciences

For a reference copy of the document with all sections, see nature.com/documents/nr-reporting-summary-flat.pdf

Ecological, evolutionary & environmental sciences study design

All studies must disclose on these points even when the disclosure is negative.

Study description	Putative SMT sequences were collected from NCBI. Sequences were vetted to identify conserved domains, and gene trees were generated. After refining the final gene tree via species tree reconciliation, the gene tree was used to produce a molecular clock. In addition, yeast knockouts were performed using S. cerevisiae strain BY4742. The presence of the C. teleta smt at the ERG6 location was screened by yeast colony PCR and subsequently grown in SC-LEU liquid media at 30°C. Lipids were extracted from yeast colonies for GC-MS analysis.
Research sample	Yeast research was performed using S. cerevisiae strain BY4742 (MATα his3Δ1 leu2Δ0 lys2Δ0 ura3Δ0)
Sampling strategy	Yeast colonies were selected on SC-LEU agar plates and grown at 30°C for ~1-2 weeks. The presence of the C. teleta smt at the ERG6 location was screened by yeast colony PCR and subsequently grown in SC-LEU liquid media at 30°C. Colonies were sampled in triplicate for lipid extraction and GC-MS analysis.
Data collection	Genetic data was collected by D.A.G., T.B. and C.M. Yeast were analyzed by P.S. and K.V. GCMS data was collected by A.S., S.O.R. , and R.E.S.
Timing and spatial scale	Yeast experiments were performed in September 2021. Yeast lipid extractions and GC-MS analysis was performed October 2021. The final round of genetic data was collected from NCBI in May 2022.
Data exclusions	All data collected was included in the analysis. In the genetic analysis, some sequences were removed from the final analysis. After the initial tree was produced, individual sequences were removed from a clade for the following reasons: (1) the sequence was redundant, meaning it was highly similar to another sequence from the same species, or (2) the sequence came from a species in a different phylum than the clade, making it a probable contaminant. Details are provided in Supplementary Figure 1
Reproducibility	Yeast samples were performed in triplicate to ensure the results were reproducible.
Randomization	No randomization was performed in this study. None of the analysis performed (phylogenetic analysis, yeast transformation, lipid

extraction) are amenable to randomization.

Blinding	No blinding was performed in this study. None of the analysis performed (phylogenetic analysis, yeast transformation, lipid extraction) are amenable to randomization.

Did the study involve field work? ☐ Yes ☒ No

Reporting for specific materials, systems and methods

We require information from authors about some types of materials, experimental systems and methods used in many studies. Here, indicate whether each material, system or method listed is relevant to your study. If you are not sure if a list item applies to your research, read the appropriate section before selecting a response.

Materials & experimental systems

n/a	Involved in the study
☒	☐ Antibodies
☒	☐ Eukaryotic cell lines
☒	☐ Palaeontology and archaeology
☒	☐ Animals and other organisms
☒	☐ Clinical data
☒	☐ Dual use research of concern
☒	☐ Plants

Methods

n/a	Involved in the study
☒	☐ ChIP-seq
☒	☐ Flow cytometry
☒	☐ MRI-based neuroimaging

Plants

Seed stocks	*Report on the source of all seed stocks or other plant material used. If applicable, state the seed stock centre and catalogue number. If plant specimens were collected from the field, describe the collection location, date and sampling procedures.*
Novel plant genotypes	*Describe the methods by which all novel plant genotypes were produced. This includes those generated by transgenic approaches, gene editing, chemical/radiation-based mutagenesis and hybridization. For transgenic lines, describe the transformation method, the number of independent lines analyzed and the generation upon which experiments were performed. For gene-edited lines, describe the editor used, the endogenous sequence targeted for editing, the targeting guide RNA sequence (if applicable) and how the editor was applied.*
Authentication	*Describe any authentication procedures for each seed stock used or novel genotype generated. Describe any experiments used to assess the effect of a mutation and, where applicable, how potential secondary effects (e.g. second site T-DNA insertions, mosiacism, off-target gene editing) were examined.*
