# Supplementary figures and images for: Common origin of sterol biosynthesis points to a feeding strategy shift in Neoproterozoic animals
Source: Nat Commun. 2023 Dec 1;14:7941. doi: 10.1038/s41467-023-43545-z (PMC10692144; doi:10.1038/s41467-023-43545-z)

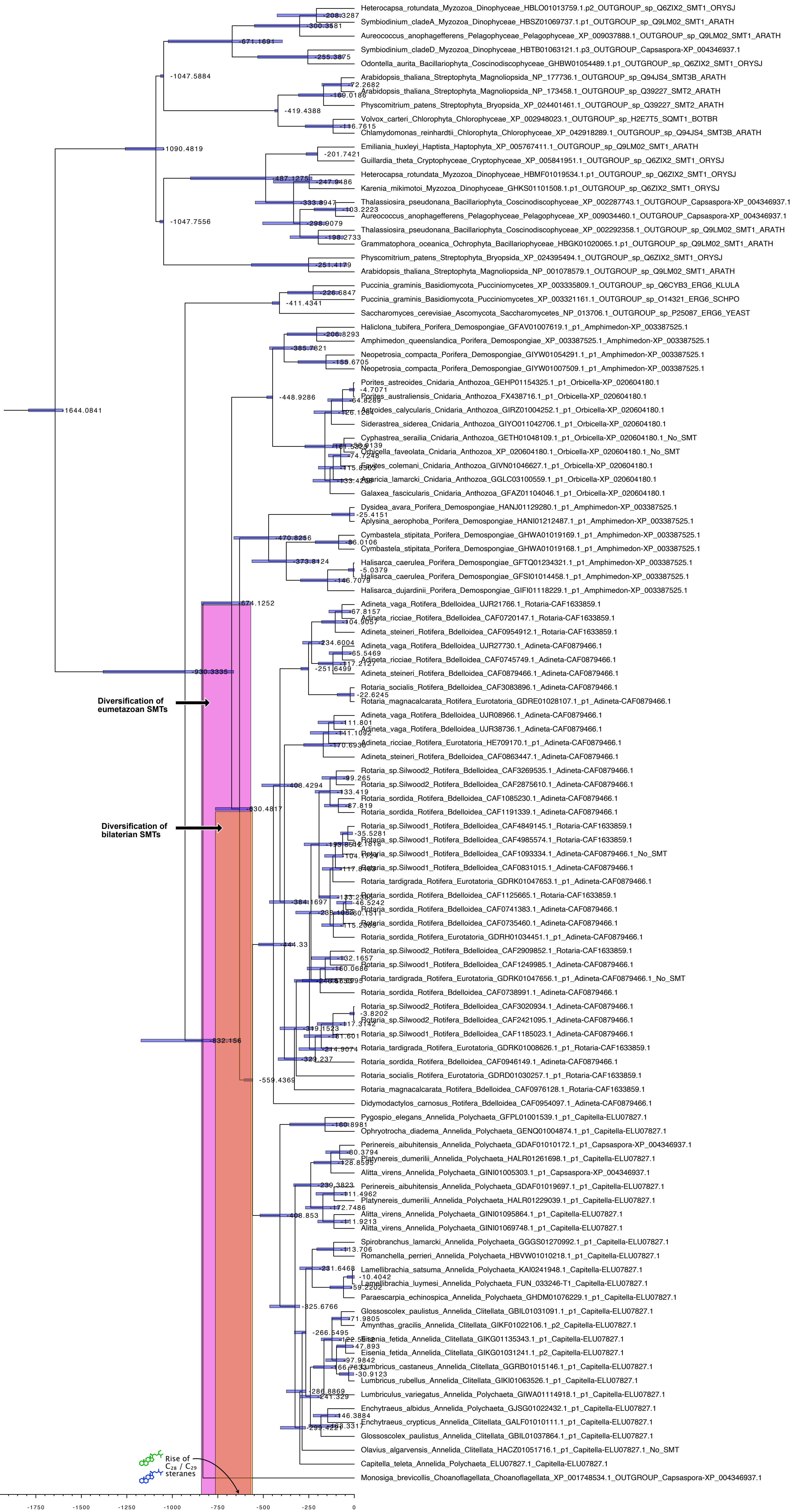

Supplement: Supplementary file 6 — Source Data [file 41467_2023_43545_MOESM6_ESM.zip › Source Data/Figure_5/12_SMT_BEAST.con.tree.pdf]
